# Exposure to Rice Straw Ash Alters Survival, Development and Microbial Diversity in Amphibian Tadpoles

**DOI:** 10.1002/ece3.71801

**Published:** 2025-07-25

**Authors:** Qing Tong, Yue‐liang Pan, Qiu‐ru Fan, Wen‐jing Dong, Xin‐zhou Long, Ming‐da Xu, Li‐yong Cui, Zhi‐wen Luo

**Affiliations:** ^1^ School of Biology and Agriculture Jiamusi University Jiamusi China; ^2^ Jiamusi Branch of Heilongjiang Academy of Forestry Sciences Jiamusi China

**Keywords:** ecological conservation, ecosystem stability, microbial diversity, microbiota, straw burning

## Abstract

Amphibians are increasingly threatened by human activities, with rice straw burning emerging as a significant yet underexplored hazard. This practice may release harmful polycyclic aromatic hydrocarbons (PAHs), disrupt ecosystems, and affect amphibians. However, the impact on tadpole microbiota and development remains unclear. This study used scanning electron microscopy (SEM) and chemical analysis to characterize straw ash toxicity, assessed rice straw aqueous extracts of ash (AEA; 0, 0.75, 1.5, 3, and 6 g L^−1^) on *Rana dybowskii* tadpoles survival, growth, and development, and analyzed skin and gut microbiota via Illumina sequencing. Within the AEA, 10 varieties of PAHs exhibited higher quantities, including acenaphthylene, acenaphthene, and anthracene. SEM revealed irregular, porous, layered ash particles. Higher AEA concentrations reduced survival, delayed development, and affected body mass. The alpha diversity of both skin and gut microbiota significantly varied among groups. Beta diversity analyses indicated substantial shifts in microbial community structure with increased AEA concentrations. Linear discriminant analysis (LEfSe) identified microbial taxa enrichment and shifts, including the increase of potentially pathogenic genera such as *Citrobacter* and *Yersinia* in high‐concentration groups. BugBase analysis showed significant phenotypic changes in microbial communities. Our findings expose rice straw ash as a silent, global toxin that disrupts amphibian microbiota, growth, and survival—redefining routine straw burning as a planetary biodiversity hazard and urging immediate, sustainable reforms to protect wetland ecosystems.

## Graphical abstract

This study examined how aqueous ash extracts (AEA) from rice straw burning impact *R. dybowskii* tadpoles, revealing that higher concentrations (up to 6 g L^−1^) over 28 d reduced survival, impaired growth, and disrupted skin and gut microbiota diversity, with polycyclic aromatic hydrocarbons (PAHs) detected in water. These microbial shifts and physiological effects underscore the ecological risks of agricultural ash pollution, advocating for sustainable practices to safeguard amphibian health and ecosystem stability.

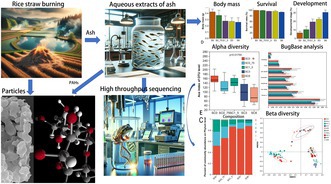

## 1 Introduction

Amphibians, one of the world's most endangered animal species, are facing previously unprecedented survival challenges (Reid et al. 2019). Compared to birds and mammals, a significantly higher proportion of amphibian species (over 40%) are in danger of going extinct, according to data from the International Union for Conservation of Nature (IUCN) (Harfoot et al. 2021). This concerning trend indicates a decline in biodiversity, ecological integrity, and overall environmental well‐being, thus underscoring the critical necessity for immediate conservation measures (Reid et al. 2019; Harfoot et al. 2021). Human activities are the main causes of the decline of amphibian species, such as climate alteration, environmental contamination, habitat destruction, introduction of alien species, and pathogen dissemination (Pabijan et al. 2020). Rice farming provides the ideal aquatic and terrestrial environments for amphibians; however, management practices (e.g., in‐field burning of rice straw) and pesticide use may have a negative impact on their health (Shuman‐Goodier et al. 2019). The symbiotic link between host and microbiota may be the first to be disrupted. Cutaneous and gut microbiota are crucial for host health, facilitating nutrient absorption, immunological resilience, physiological function, and pathogen defense (Zhu et al. 2024; Flechas et al. 2019). Rice farming management practices can pose considerable environmental risks and ecological disturbances, negatively impacting amphibians and their essential skin and gut microbiota, hence underscoring conservation issues (Jiménez and Sommer 2016).

Agricultural fires significantly affect species diversity and density, although their impact on amphibians is insufficiently studied (Mitchell et al. 2023). In 2018, worldwide rice production exceeded 0.76 billion tons, whereas straw production surpassed 1.10 billion tons (Singh et al. 2021). Owing to the diminishing need for straw as fuel and fodder, coupled with the reduced interval between crop sowing and rising costs, farmers choose the economically advantageous practice of field straw incineration (Seglah et al. 2020). Furthermore, the straw gathered after the autumn harvest is frequently incinerated again in the subsequent autumn and spring seasons, rendering our study extremely environmental correlation (Long et al. 2025). Despite the adverse effects on human health, ecology, and soil quality, the tendency to engage in straw burning persists (Singh, Chowdhary, and Sharma 2024). Around the globe, only 20% of the produced straw is utilized, while over 100 million tons of straw are burnt each year (Singh, Sharma, et al. 2024). Most research focuses on how burning straw affects biodiversity, climate change, air pollution, soil degradation, and visual pollution (Chen et al. 2017). Numerous detrimental effects on soil fertility, ecosystems, air quality, and climate have been shown by research (Shi et al. 2014). When straw is not burned sufficiently, carcinogens such as polycyclic aromatic hydrocarbons (PAHs) are released, endangering the integrity of the environment (Rajput et al. 2023). However, the effects of burning straw on ecosystems and water pollution have been overlooked (Zhang et al. 2022). Rice straw burning can alter the chemistry of water and endanger amphibian feeding chains, habitats, and health (Mahood et al. 2020). Burning straw causes nitrogen depletion, eutrophication, and deterioration of water quality, which encourage the growth of germs and viruses (Linquist et al. 2014). Ash released during floods or rains can kill aquatic life and increase the vulnerability of water ecosystems to species invasion (Nunes et al. 2017; Gonino, Figueiredo, et al. 2019). Sublethal doses of sugarcane ash (0.8 g L^−1^) have been found to slow down fish behavior responses after a short‐term (24 h) exposure (Yofukuji et al. 2021). Currently, there is a lack of research on the impact of rice straw burning on amphibians, particularly regarding its effects on early developmental stages.

Amphibians' skin microbiota are critical to their health, which prevents infections and supports immunological (Bates et al. 2022). Due to their sensitivity to changes in the environment, these microbiota have a major impact on amphibian health and growth (Hernández‐Gómez and Hua 2023). Rainy runoff containing rice straw ash may threaten the health and growth of amphibians by introducing toxins, disrupting pH and nutrient cycles, and inducing osmotic and physical stress on their skin microbiota (Gomez Isaza et al. 2022). Rice straw ash alters water pH, disturbs amphibian skin microbiota by promoting harmful bacteria, thereby directly affecting microbiota composition (Arunrat et al. 2023). PAHs emitted by ash adversely affect skin microbiota, ultimately leading to a significant decrease in microbiota diversity, which is crucial for amphibian resilience (Barathi et al. 2023). In human subjects from China, prolonged exposure to PAHs altered the makeup and function of the cutaneous microbiota (Leung et al. 2020). Alterations in water chemistry caused by ash disrupt the nutrient cycling essential for the maintenance and function of skin microbiota, thereby disturbing microbial homeostasis (Gomez Isaza et al. 2022). Ash particles physically damage microbial biofilms on frog skin, increasing the susceptibility to pathogen invasions that disrupt microbial symbiosis (Afonso et al. 2022). Wildfire ash exposure altered the skin microbiota of *Rana dybowskii* tadpoles, and scanning electron microscopy (SEM) revealed irregular, porous ash particles that might have physically disrupted microbial biofilms and increased susceptibility to pathogens (Tong et al. 2025). Amphibians are especially susceptible to environmental pollution due to their unique characteristics (Coelho et al. 2022). However, there is a lack of studies examining how straw ash affects the skin microbiota of amphibians like tadpoles (Gomez Isaza et al. 2022).

Rice straw ash may adversely affect amphibians, especially gut microbiota, while data are scarce (Han et al. 2018). The balance of gut microbiota is essential for health, facilitating digestion, vitamin synthesis, immunoregulation, pathogen defense, detoxification, and maintaining gut function (Ya et al. 2019). Harmful chemical compounds in rice straw ash may disrupt the gut microbiota of amphibians, resulting in dysbiosis by eliminating or suppressing some beneficial microbial species (Bhattacharyya et al. 2020). Research has revealed that exposure to PAHs alters the gut microbiota and metabolite levels in Atlantic killifish (Redfern et al. 2021). This dysbiosis diminishes the population of beneficial bacteria and may negatively impact the digestive capacity and immunity of amphibians (Dawood et al. 2019). Moreover, the burning of rice straw in proximity to freshwater ecosystems can significantly modify the physicochemical characteristics of the water, thereby indirectly influencing the gut microbiota of amphibians by altering water quality and the availability of food resources (Elbasiouny et al. 2020). Such variations can influence the tadpoles' feeding behavior and interfere with their normal digestive processes and nutrient absorption, thereby affecting the composition and functionality of the intestinal microbiota (Huang et al. 2018). Harmful substances present in ash may be directly ingested by amphibians through their diet, potentially causing detrimental or toxic impacts on their gut microbiota (Chen et al. 2024). For example, exposure to wildfire ash extracts significantly disrupted the gut microbiota of *R. dybowskii* tadpoles, highlighting the ecological risk posed by ash‐associated pollutants such as PAHs and heavy metals (Tong et al. 2025). Current research on straw burning focuses on ecotoxicology; however, it lacks studies about its effects on the gut microbiota of amphibians (Gonino, Figueiredo, et al. 2019; Gonino, Branco, et al. 2019; Oliveira et al. 2018).

An important frog in Northeast China, *R. dybowskii* , reproduces in the spring, when its tadpoles are most active (Tong et al. 2020). In the distribution region of *R. dybowskii* , extensive rice fields are cultivated, and the incineration of spring stalks is prevalent for the disposal of rice straw (Long et al. 2025; Singh et al. 2020). Frog tadpoles may be disturbed by burning rice straw in the spring (Baba et al. 2023; Woodrow et al. 2019). Our earlier studies indicated that both wildfire and rice straw ash disrupt the skin and gut microbiota of hibernating adult frogs and reduce their survival (Dong et al. 2024; Xu et al. 2024). Tadpoles, at a crucial developmental phase characterized by fast growth and underdeveloped immune systems, may exhibit heightened sensitivity to contaminants like rice straw ash (Long et al. 2025). In contrast to hibernating adult frogs, they engage in active feeding, hence increasing their exposure to environmental contaminants (Dong et al. 2024). Their health and microbiota alterations render them significant markers of ecological consequences in both aquatic and terrestrial ecosystems (Turriago et al. 2022). Using high‐throughput sequencing and frog tadpole samples, this study examines the effects of varying concentrations of rice straw ash on the survival, development, and diversity of their skin and gut microbiota (Tong et al. 2020). We hypothesize that exposure to varying concentrations of rice straw ash in aquatic environments will increase tadpole mortality, alter the composition and diversity of skin and gut microbiota, disrupt tadpole development, and induce changes in microbial phenotypes in *R. dybowskii* across a 28‐days testing period.

## 2 Materials and Methods

### 2.1 Experimental Animals

*Rana dybowskii* were collected from Luobei County (47.5789 N, 130.3794E) in Heilongjiang Province, China. Both sexes of frogs were collected from a hibernation pond when they began fasting in November due to falling temperatures. The frogs, with a weight of 20.48 ± 1.16 g, were under optimal conditions. Hibernation transpired in university laboratory wintering ponds over the winter season. Tadpoles were created in March after mating and spawning in Jiamusi University laboratory. Through cultivation, the tadpoles reached Gosner stage 20 (G20), with an average weight of 0.12 ± 0.01 g (Park et al. 2023). On the 28th day, tadpoles progressing to stage G26 were sent to the laboratory for additional examination.

### 2.2 Rice Straw Ash

The ash was obtained from the burning of rice straw in Zhaoyuan County (45.6990 N, 124.2460E), Heilongjiang Province, China, and the black ash formed during the combustion process. Ash was promptly gathered following the combustion and was treated with defined techniques to guarantee sample uniformity. To account for the non‐uniformity of the ash layer, 30 sites within the straw burning zones were randomly selected for sampling (Coelho et al. 2022; Santos et al. 2023a). At each designated site, a 50 × 50 cm^2^ plot was set up. The entire ash layer was meticulously collected with a spoon and brush, then passed through a 2 mm sieve for filtration (Santos et al. 2023a). Ash samples, free from soil contamination, were collected, placed in plastic bags, mixed together, and stored at −18°C in a dark laboratory to prevent bioactivity before conducting the ecotoxicological analysis (Santos et al. 2023a).

### 2.3 Aqueous Extracts of Ashes

The generation of rice straw aqueous extracts of ash (AEA) for ecotoxicity evaluations requires a careful and systematic methodology (Santos et al. 2023a). The levels of ambient samples and AEA were selected to replicate actual levels found in freshwater settings amid peak influx and dilution in lotic systems (Mirč et al. 2023). The maximum concentration (10 g L^−1^) was established as per other investigations (Santos et al. 2023b; Jesus et al. 2023; González et al. 2023). For example, in toxicity experiments on *Daphnia magna* with ash from Australian wildfires, it was shown that at a concentration of 25 g L^−1^, the 24 h mortality rate was 100%, while at lower concentrations (6.25 and 10 g L^−1^), the mortality rates were 10% and 75%, respectively (Harper et al. 2019). Our recent research also indicated that different concentrations (0, 1.25, 2.5, 5, and 10 g L^−1^) of straw ash and wildfire ash exhibited significant toxicity to *R. dybowskii* (Dong et al. 2024; Xu et al. 2024). Before starting our experiment, we conducted a preliminary acute toxicity test. We reared the tadpoles to the G20 stage and subsequently conducted acute toxicity testing utilizing AEA concentrations of 2, 4, 6, 8, and 10 g L^−1^. The 48 h survival rates of the tadpoles at doses of 2, 4, and 6 g L^−1^ were all 100%. Mortality occurred at elevated amounts. Therefore, we set concentrations at 0, 0.75, 1.5, 3, and 6 g L^−1^ to obtain viable samples for a 28‐days investigation into the chronic effects of ash on tadpole growth and skin and gut microbiota.

A precise and controlled method was used to analyze the ecotoxicity of AEA (Santos et al. 2023a). To prepare a 10 g L^−1^ solution of straw ash, 500 g of straw ash was dissolved in 50 L of water (Santos et al. 2023a). The mixture was well agitated and settled for 2 days, guaranteeing a consistent dispersion of ash particles. After that, the clarified supernatant in the desired concentration of 10 g L^−1^ was removed. AEA at these concentrations was prepared precisely and synchronized with the initiation of the assay and each medium alteration thereafter (Santos et al. 2023a). Samples of 10 g L^−1^ AEA filtered through a 0.45 μm syringe were analyzed for PAHs at an external laboratory and confined to 16 PAHs recognized by the United States Environmental Protection Agency (EPA) (Afonso et al. 2022). A technique for analyzing 16 trace PAHs in irrigation water was established on the basis of liquid–liquid extraction, silica gel solid‐phase extraction purification, and gas chromatography–mass spectrometry (GC–MS). The sample preparation protocol for this technique is well‐defined and provides good measuring precision. Effective mitigation of complex matrix interferences is achieved by MS operating in the selective ion scanning mode. This technique is appropriate for identifying trace PAHs in irrigation water with complicated matrices, including ash‐laden water (Chuang‐Li and Wei 2023; Rascón et al. 2018). The chosen samples were studied using SEM to investigate the ash's distinctive surface features. The samples were mounted on aluminum stubs with glue and then gold‐plated under pressure. The samples were finally examined using SEM (model: Sigma with Gemini column, Carl Zeiss, the United States).

### 2.4 Experimental Design

A 28‐days experiment was performed to evaluate the effects of AEA on the body mass, survival rate, development, and skin and gut microbiota of *R. dybowskii* . Five treatment groups were set: S0 (control, 0 g L^−1^), S0_75 (0.75 g L^−1^), S1_5 (1.5 g L^−1^), S3 (3 g L^−1^), and S6 (6 g L^−1^). Each group was tested in triplicate, with 35 tadpoles in each container.

The assessment of cutaneous microbiota (C) and gut microbiota (G) both involved five treatment groups: SC0 (control, 0 g L^−1^), SC0_75 (0.75 g L^−1^), SC1_5 (1.5 g L^−1^), SC3 (3 g L^−1^), and SC6 (6 g L^−1^); SG0 (control, 0 g L^−1^), SG0_75 (0.75 g L^−1^), SG1_5 (1.5 g L^−1^), SG3 (3 g L^−1^), and SG6 (6 g L^−1^). Three duplicates were set for each group, with 35 tadpoles per container. Eight or nine samples per group were collected for microbiota analysis, with three tadpoles picked from each container and labeled from 1 to 9.

Each bucket with 40 L of an AEA mixture contained 35 tadpoles. The bucket was positioned in a laboratory with windows and air conditioning, ensuring it was not directly exposed to sunlight or the airflow from the air conditioning, so maintaining the water temperature at 15.9°C ± 1.8°C, and utilized the temperature recorder (ZDR‐20) to monitor the temperature in real time (Mirč et al. 2023). The tadpoles received daily nourishment of fish food flakes and rabbit pellets (Mirč et al. 2023; Hernández‐Gómez et al. 2020). Lighting was configured for a 12‐h light/dark cycle. Every 5 days of the 28‐days experiment, the AEA solutions were refilled. The survival of the tadpoles was observed daily, and the number of dying individuals was documented. The tadpoles were collected to examine their development and morphology in water, with the hind limb buds enlarged (Nicieza 1999). To determine phases G22–G25, the alterations were analyzed and contrasted with conventional developmental pictures (Schotthoefer et al. 2003). Using a long pipette, dead tadpoles were removed, and waste was removed from each container, preserving the water's clarity.

Tadpole samples were quickly transported to the Jiamusi University lab on Day 28, where skins were immediately dissected. In euthanization, tadpoles were immersed in a glass desiccator that contained a mixture of tricaine methanesulfonate (MS‐222) and alcohol for sedation (Hernández‐Gómez et al. 2020; Shu et al. 2022). Death was defined as the lack of reaction to stimuli and signs (Forgione and Brady 2022). After rinsing the tadpoles with ultrapure water, collected skin samples (excluding tail and toe tissues) for 16S rRNA gene sequencing. After euthanasia, the gastrointestinal tract of each tadpole was carefully separated from the body, commencing at the anal region and proceeding upwards, excluding the stomach. 10 min after death, the small and large intestines were removed. Carefully gathered the intestinal contents into a sterile 5 mL receptacle. Each specimen was collected with a new set of sterile tweezers to prevent cross‐contamination. Each intestinal specimen was preserved in a sterile vial and subsequently frozen at −80°C.

### 2.5 DNA Extraction and PCR Amplification

Microbial DNA from skin and gut microbiota was extracted by homogenizing materials and utilizing a FastDNA spin kit for soil (MP Biomedical, the United States) according to the specified technique. The quality of DNA was evaluated using 1% agarose gel electrophoresis, while the DNA concentration and A260/A280 ratio were measured with a NanoDrop 2000 spectrophotometer (Thermo Scientific, the United States). The V3‐V4 regions of bacterial 16S rRNA genes were amplified utilizing the primers 338F (5′‐ACTCCTACGGGAGGCAGCAG‐3′) and 806R (5′‐GGACTACHVGGGTWTCTAAT‐3′). The PCR protocol consisted of denaturation at 95°C for 3 min, followed by 27 cycles of 30 s at 95°C for denaturation, 30 s at 55°C for annealing, and 45 s at 72°C for extension, concluding with a final extension at 72°C for 10 min. The PCR mixture included 4 μL of 5 × FastPfu buffer, 0.4 μL of FastPfu polymerase, 2 μL of 2.5 mM dNTPs, 10 ng of template DNA, 0.8 μL of each 5 μM primer, and sterile ddH_2_O to a final volume of 20 μL. Axygen Biosciences' AxyPrep DNA gel kit was used to purify the PCR results after they had been separated using a 2% agarose gel. A Promega QuantiFluor‐ST test kit was used to quantify DNA.

### 2.6 Illumina MiSeq

Following the standardization of amplicon levels, the samples underwent quality assessment, quantification, and sequencing utilizing paired‐end 2 × 300 reads on an Illumina MiSeq platform (Illumina, the United States). Microbiota sequence data are available in the NCBI SRA database under accession numbers PRJNA1050065, PRJNA1058650, PRJNA1058652, PRJNA1058654, PRJNA1050068, PRJNA1050064, PRJNA1058657, PRJNA1058658, PRJNA1058659, and PRJNA1050067.

### 2.7 Processing of Sequencing Data

Raw fastq files underwent demultiplexing, quality filtering by Trimmomatic, then merging through FLASH. Sequences of 300 base pairs (bp) were cut in areas where a 50 bp window had a mean quality score below 20, retaining only those sequences beyond 50 bp for subsequent analysis. Sequences were generated from overlaps above 10 bp, while those that could not be assembled were discarded. Furthermore, sequences with barcode errors, more than two primer mismatches, or ambiguous bases were discarded. Operational taxonomic units (OTUs) were defined at 97% similarity using uPARs 7.1 and were purified of chimeras with UCHIME. All 16S rRNA sequences were taxonomically categorized utilizing the Silva (SSU138) database, with a confidence threshold of 70%.

### 2.8 Ecological and Statistical Analysis

A 28‐days experiment was conducted to investigate the impact of AEA on the body mass (Day 17), development (Day 28), and survival rate (Day 28) of *R. dybowskii* among five groups. The consolidation of phases G22‐G23 and G24‐G25 streamlines analysis and highlights shared physical developmental traits (Xu et al. 2024). Differences among multiple groups were assessed utilizing analysis of variance (ANOVA) and Tukey's honestly significant difference (HSD) test for subsequent pairwise comparisons. Rarefaction curves were generated using Mothur 1.30.2 (https://mothur.org/wiki/calculators/) to assess the alpha diversity metrics of skin and gut microbiota, encompassing the abundance‐based coverage estimator (ACE), Chao, Shannon, and observed species (Sobs) indices (Hadizadeh et al. 2017). Data were analyzed using the FDR‐corrected Kruskal‐Wallis H (KWH) test and multiple comparisons. Only *p* < 0.05 was reported. The tadpoles skin and gut microbiota, present in 90% of samples and comprising over 0.1% of total sequencing data, were deemed dominant. A Venn diagram was created using R software version 3.3.1 to identify unique and overlapping OTUs.

After calculating beta diversity distance matrices with Qiime (http://qiime.org/scripts/assign_taxonomy.htm), non‐metric multidimensional scaling (NMDS) analysis and pertinent graphing were conducted using R vegan 2.4.3 (Knights et al. 2011). To evaluate the effect of straw ash at designated concentrations on community clustering and population dispersion, analyses of similarity (ANOSIM) and multivariate non‐parametric variance (Adonis, with 999 permutations) were employed, utilizing both Bray–Curtis dissimilarity and weighted UniFrac distance derived from OTU‐level tables.

Dominant species and their relative abundances at designated taxonomic levels in each sample were identified utilising R 3.3.1, with data obtained from the folder tax_summary_a. Relative abundance was calculated as the ratio of sequence counts for each taxon to the total sequence counts within each sample. The differences in relative abundance among groups were evaluated using the KWH test and adjusted for multiple comparisons with the Benjamini‐Hochberg false discovery rate. Differences at adjusted *p* < 0.05 were displayed.

By combining significance with biological relevance, distinct phyla and genera were discovered using linear discriminant analysis (LDA) effect size (LEfSe, LDA > 4) (Lian et al. 2019). In order to identify and predict the high‐level phenotypic characteristics present in samples, BugBase for assessing microbiota was used (Lucas et al. 2018). BugBase normalizes OTUs with estimated 16S rRNA gene copy counts to describe the microbiome using precomputed data. The KWH test was used to assess the relative abundance variance across groups at the significance level *p* < 0.05.

## 3 Results

### 3.1 PAHs and Particle Observation

For every chemical, the measured concentrations and the limit of quantification (LOQ) were provided. Acenaphthene (ACE, 1.21 × 10^−4^), Acenaphthylene (ACY, 6.50 × 10^−5^), Anthracene (ANT, 1.52 × 10^−4^), Benzo(a)anthracene (BaA, 1.60 × 10^−5^), Chrysene (CHR, 7.90 × 10^−5^), Fluoranthene (FLT, 1.29 × 10^−4^), Fluorene (FLU, 2.09 × 10^−4^), Naphthalene (NAP, 8.83 × 10^−4^), Phenanthrene (PHE, 6.17 × 10^−4^), and Pyrene (PYR, 1.75 × 10^−4^) were among the compounds that significantly exceeded the corresponding LOQs (μg mL^−1^) (Table 1). A least existence was indicated by several chemicals; nonetheless, they were below LOQs (Table 1).

**TABLE 1:** Polycyclic aromatic hydrocarbons (PAHs) concentrations in 10 g L^−1^ rice straw aqueous extracts of ashes (AEA).

Polycyclic aromatic hydrocarbons (PAHs)	LOQ (μg/mL)	Straw ash (μg/mL)
Acenaphthene (ACE)	1.61 × 10^−5^	1.21 × 10^−4^
Acenaphthylene (ACY)	7.80 × 10^−6^	6.50 × 10^−5^
Anthracene (ANT)	8.72 × 10^−6^	1.52 × 10^−4^
Benzo(a)anthracene (BaA)	9.13 × 10^−6^	1.60 × 10^−5^
Benzo(a)pyrene (BaP)	2.15 × 10^−3^	< 2.15 × 10^−3^
Benzo(b)fluoranthene (BbF)	6.49 × 10^−4^	< 6.49 × 10^−4^
Benzo(g,h,i)perylene (BghiP)	2.99 × 10^−5^	< 2.99 × 10^−5^
Benzo(k)fluoranthene (BkF)	7.27 × 10^−4^	< 7.27 × 10^−4^
Chrysene (CHR)	8.27 × 10^−6^	7.90 × 10^−5^
Dibenzo(a,h)anthracene (DahA)	4.17 × 10^−5^	< 4.17 × 10^−5^
Fluoranthene (FLT)	2.92 × 10^−6^	1.29 × 10^−4^
Fluorene (FLU)	9.83 × 10^−6^	2.09 × 10^−4^
Indeno(1,2,3‐cd)pyrene (IcdP)	2.32 × 10^−5^	< 2.32 × 10^−5^
Naphthalene (NAP)	8.40 × 10^−6^	8.83 × 10^−4^
Phenanthrene (PHE)	6.24 × 10^−6^	6.17 × 10^−4^
Pyrene (PYR)	2.72 × 10^−6^	1.75 × 10^−4^

Abbreviation: LOQ, limit of quantification.

**FIGURE 1:**
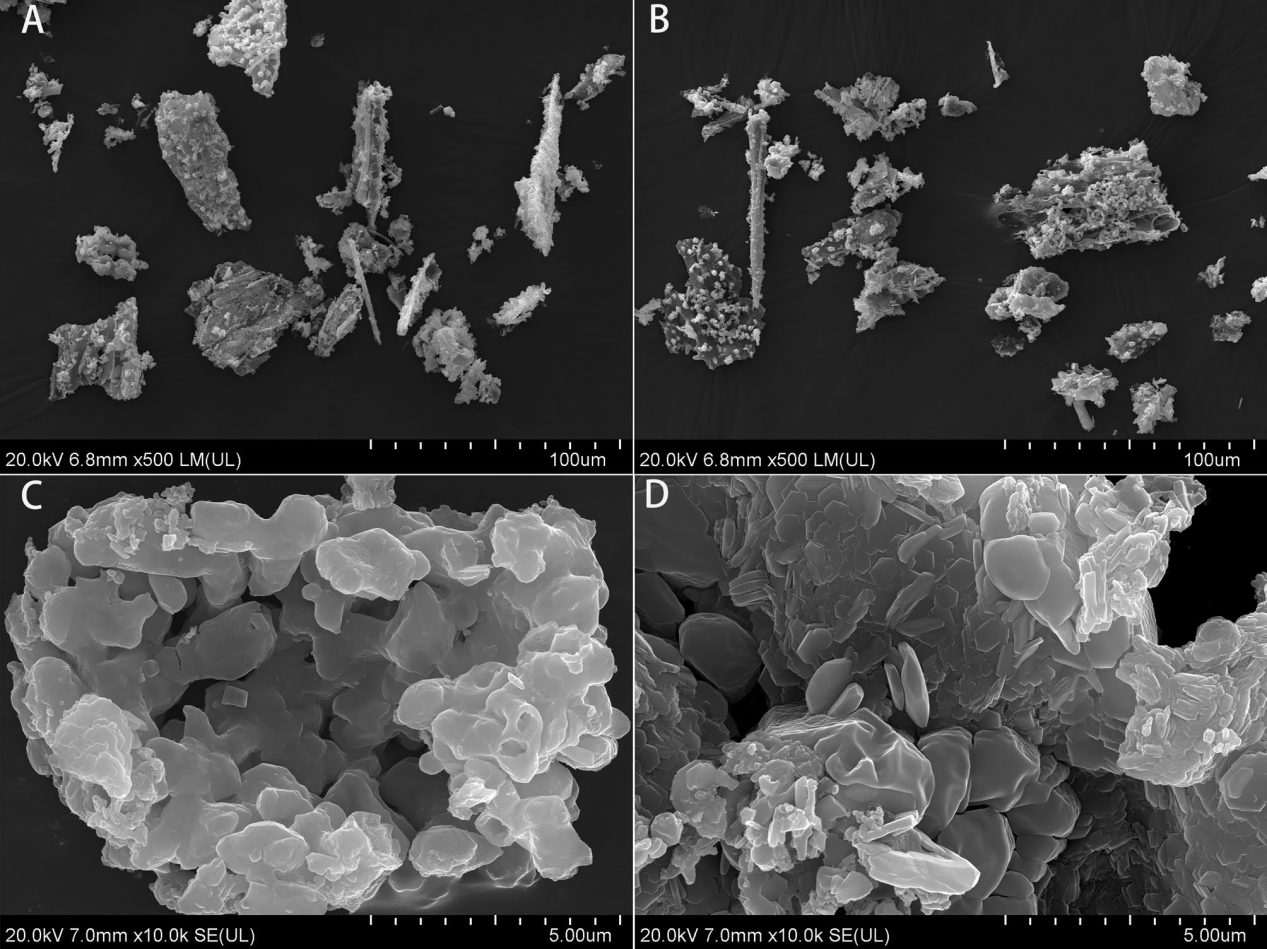
Scanning electron microscopy (SEM) analysis of straw ash. SEM reveals the post‐combustion ash particle morphology and structure at varying magnifications. At a 500‐fold magnification, (A and B) illustrate the overall morphology and surface textures of two separate particle assemblies. At a 10,000‐fold magnification, (C and D) provide a comprehensive analysis of the microstructural complexities of a solitary particle aggregate.

The image at 500× magnification revealed the overall morphology and surface texture of straw ash particles, which exhibited irregular shapes with shattered edges or angular and elongated forms (Figure 1A,B). The rough and fractured surfaces of these ash particles, characteristic of post‐combustion ash, suggested exposure to elevated temperatures resulting in surface disturbance. At 10,000× magnification, ash aggregates exhibited a layered, crystal‐like microstructure with significant porosity, indicating the adhesion present on these surfaces (Figure 1C,D).

### 3.2 Body Mass, Survival Rate, and Development Stage

**FIGURE 2:**
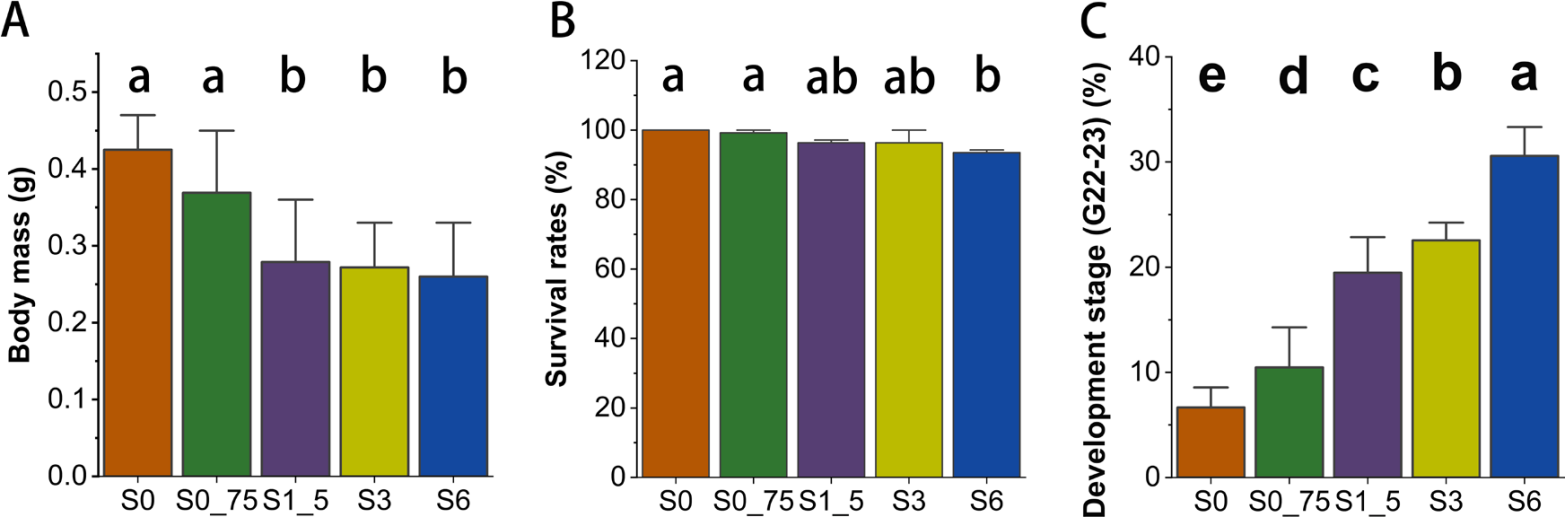
Differences in body mass, survival, and development stage among five treatment groups. Significant differences in body mass (A), survival (B), and developmental stage (C) among the five groups (S0, S0_75, S1_5, S3, and S6). Panel C presents the developmental progress of *R. dybowskii* tadpoles exposed to rice straw ash aqueous extracts for 28 days, evaluated based on the Gosner staging system. The developmental stage of five groups is expressed as the percentage of development in G22‐23, while others belong to G24‐G25 (*n* = 35).

The study indicated a significant difference in body mass among groups (ANOVA, *p* < 0.05), with groups S0 and S0_75 exhibiting higher mean values (Tukey HSD test, *p* < 0.05) (Figure 2A). The average values of groups S1_5, S3, and S6 were similar and exhibited no significant differences (Tukey HSD test, *p* > 0.05; Figure 2A).

ANOVA revealed significant differences in survival rates among the five groups (*F* = 5.51, *p* = 0.013). The subsequent Tukey HSD test revealed significant differences between groups S0 and S6, as well as between S0_75 and S6 (*p* < 0.05), while no significant differences were seen in other group comparisons (*p* > 0.05). This indicated that groups S0 and S0_75 exhibit considerably higher survival rates compared to group S6 (*p* < 0.05; Figure 2B).

The consolidation of phases G22‐G23 and G24‐G25 streamlines analysis and highlights shared physical developmental traits. Elevated levels of G22‐G23 were noted at increased concentrations, with ANOVA revealing significant differences across the groups (*F* = 33.78, *p* < 0.01). Every pair within the groups S0, S0_75, S1_5, S3, and S6 exhibited a significant difference (Tukey HSD test, *p* < 0.05; Figure 2C).

### 3.3 Alpha Diversity and Shared Microbiota

**FIGURE 3:**
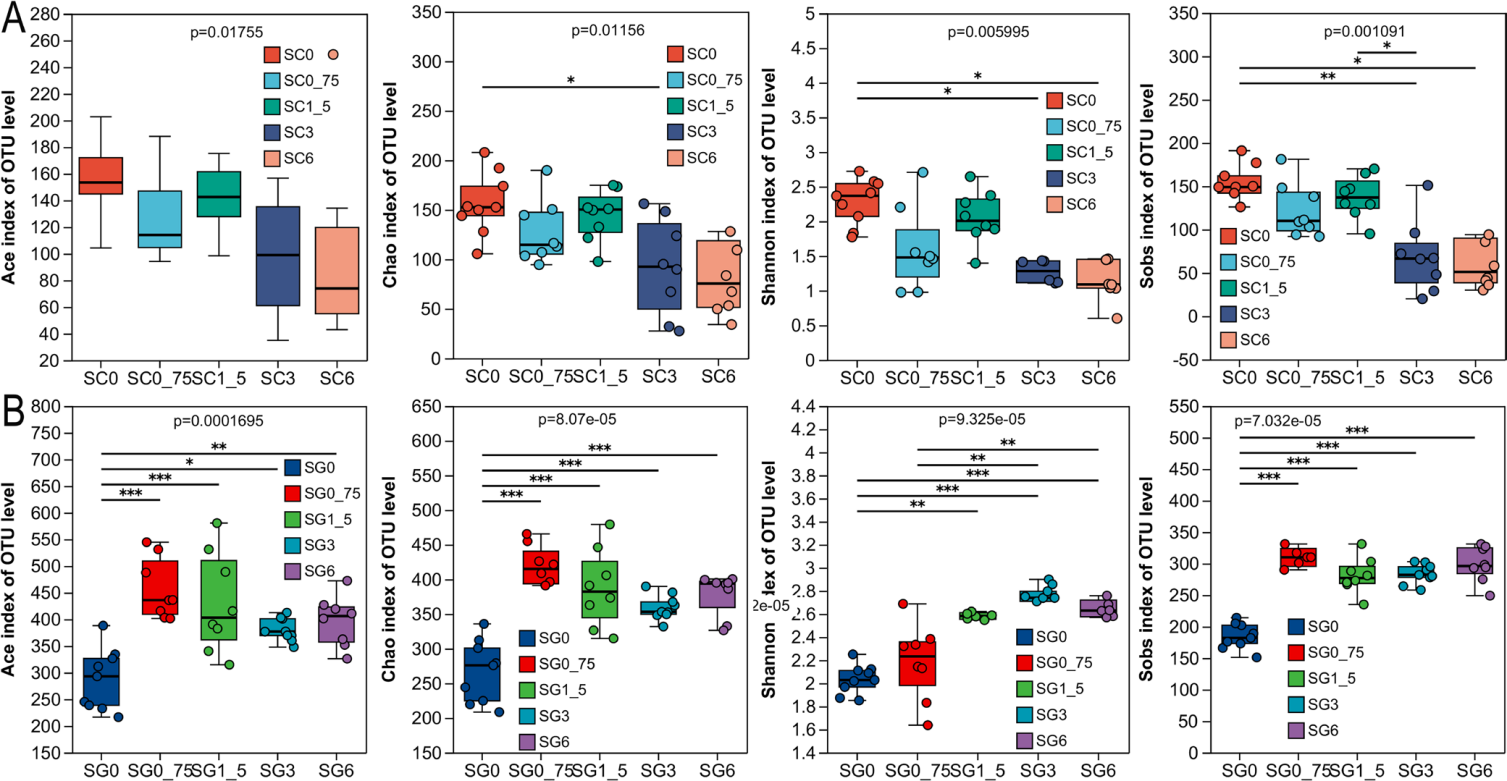
The alpha diversity of *R. dybowskii* tadpoles' skin and gut microbiota in five treatment groups. A comparative analysis of skin (A) and gut (B) microbiota alpha diversity in tadpoles exposed to five treatment concentrations (SC0, SG0, SG3: *N* = 9, SC0_75, SC1_5, SC3, SC6, SG0_75, SG1_5, SG6: *N* = 8) straw ash conditions. Analyses utilized ACE, Chao, Shannon, and Sobs indices. Significance levels are indicated: *, **, and *** for *p* values falling in 0.01 < *p* < 0.05, 0.001 < *p <* 0.01, and *p* < 0.001, respectively.

Shannon curves stabilize with increased sequencing, suggesting adequate sampling (Figure S1A,C). The plateau on rarefaction curves indicated enough sequencing depth and species abundance across samples (Figure S1B,D). ACE, Chao, Shannon, and Sobs indices all significantly differed across the five skin groups (KWH test, *p* < 0.05; Figure 3A). The above four indices varied notably among groups SG0, SG0_75, SG1_5, SG3, and SG6 (KWH test, *p* < 0.05; Figure 3B). Group SG0 exhibited the lowest diversity (KWH test, *p* < 0.05; Figure 3B).

According to the Venn diagram, 140 OTUs were found to be shared by all five gut groups (Figure S2A). Groups SG0, SG0_75, SG1_5, SG3, and SG6 had 81, 142, 99, 97, and 166 unique OTUs, respectively (Figure S2A). The five skin groups shared 94 OTUs (Figure S2B). Groups SC0, SC0_75, SC1_5, SC3, and SC6 displayed 170, 89, 126, 19, and 102 unique OTUs, respectively (Figure S2B).

### 3.4 Beta Diversity

**FIGURE 4:**
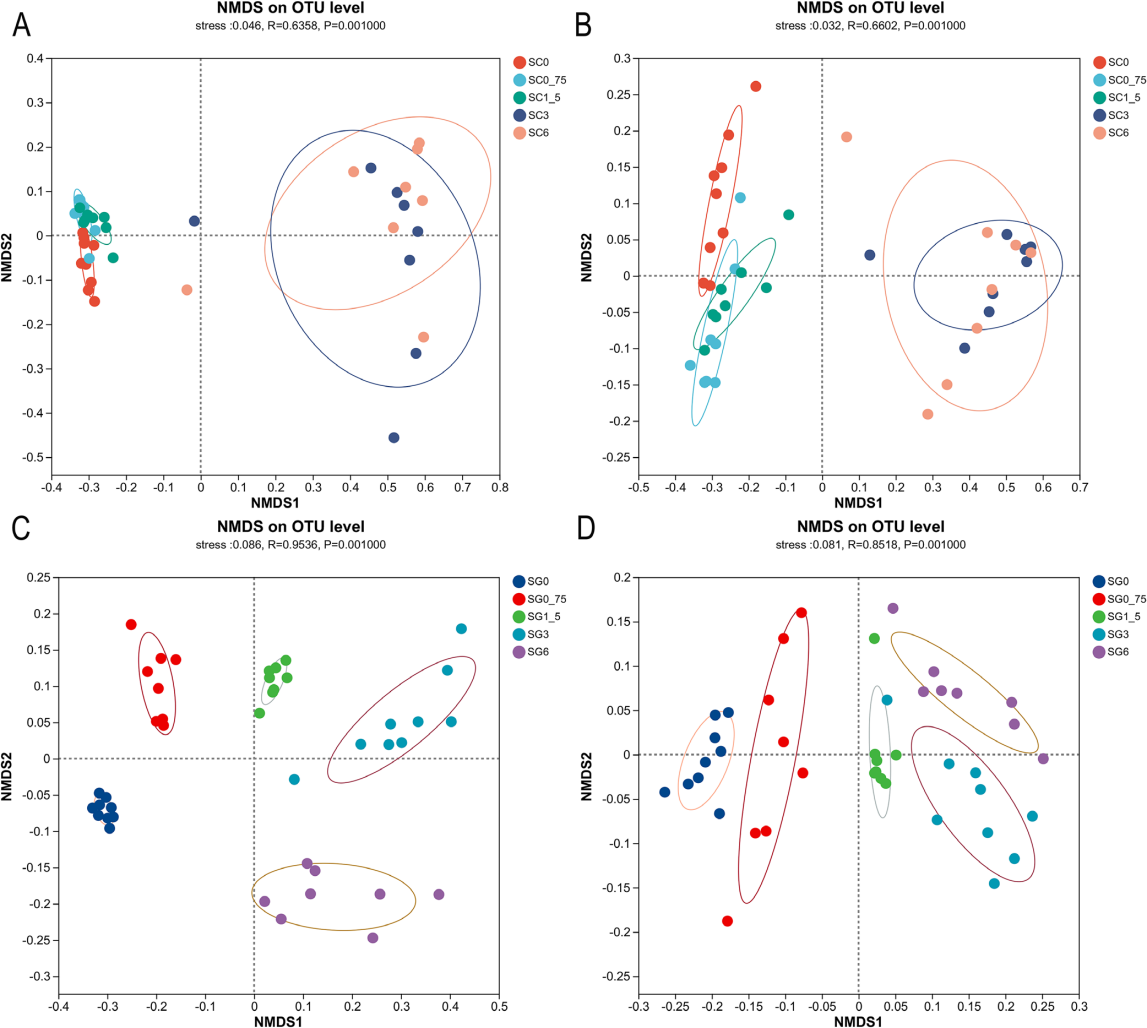
Assessing aqueous extracts of rice straw ash impact on beta diversity of tadpole microbiota with non‐metric multidimensional scaling (NMDS). To analyse the impact of aqueous extracts of rice straw ash at different concentrations on community structure and dispersion, data at the OTU level were utilized, employing the Bray–Curtis dissimilarity index (A and C) and weighing UniFrac distance (B and D). NMDS plots illustrate samples from the skin (A and B) and gut (C and D).

The Bray–Curtis dissimilarity matrix (Adonis: *R* ^2^ = 0.860, *p* = 0.001; ANOSIM: statistic = 0.571, *p* = 0.001) and weighted UniFrac distances (Adonis: *R* ^2^ = 0.858, *p* = 0.001; ANOSIM: statistic = 0.572, *p* = 0.001) significantly differed among the five skin groups (Figure 4A,B). Samples from SC0, SC0_75, SC1_5 clustered severely and were closely spaced (Figure S3A,B). Samples from SC3 and SC6 were dispersed and closely spaced (Figure S3A,B).

The Bray–Curtis dissimilarity matrix (Adonis: *R* ^2^ = 0.796, *p* = 0.001; ANOSIM: statistic = 0.600, *p* = 0.001) and weighted UniFrac distance (Adonis: *R* ^2^ = 0.803, *p* = 0.001; ANOSIM: statistic = 0.604, *p* = 0.001) metrics were significantly different in microbiota composition across the five gut groups (Figure 4C,D). Samples from the five groups clustered remarkably, with close spacing. The closer distances between groups were related to larger differences in concentration (Figure S3A,B).

### 3.5 Composition and Differences in Skin and Gut Microbiota

**FIGURE 5:**
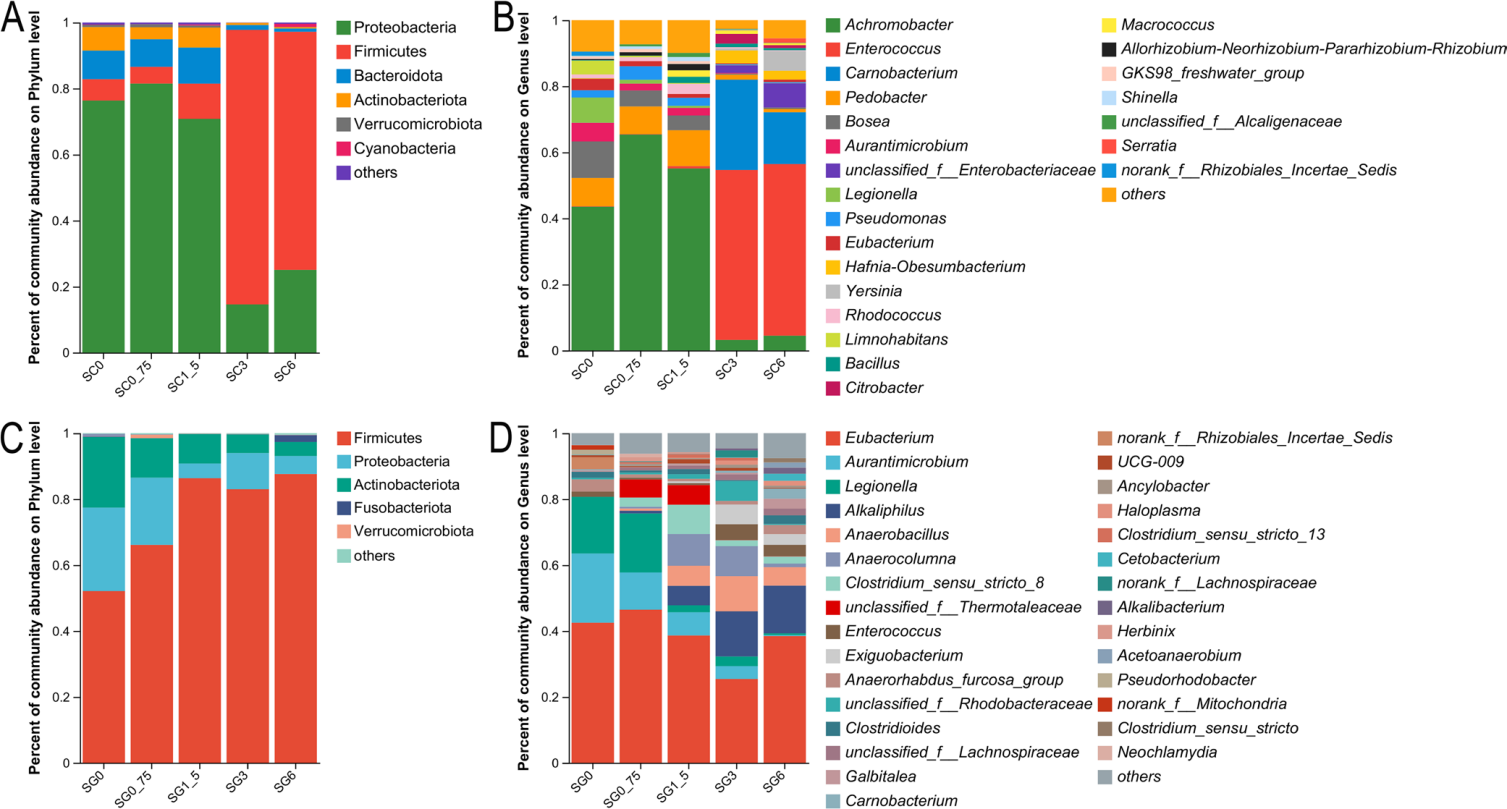
Characteristics of tadpole skin and gut microbiome composition under five treatment aqueous extracts of rice straw ash concentration levels. This study utilized bar charts to analyze microbiota at the phylum (A and C) and genus (B and D) levels in skin (A and C) and gut (B and D) microbiota among five concentrations (SC0, SG0, SG3: *N* = 9, SC0_75, SC1_5, SC3, SC6, SG0_75, SG1_5, SG6: *N* = 8). The charts only display phyla and genera with a relative abundance of over 1% in at least one sample.

The phyla in the skin microbiota were dominated by Proteobacteria, Firmicutes, and Bacteroidetes in all groups (Figure 5A). Comparative analysis revealed 12 of the 26 phyla significantly differed across the five skin groups (KWH test, *p* < 0.05). Group SC0 showed significant enrichment of *Achromobacter* (43.50%) and *Bosea* (11.03%). SC0_75 was dominated by *Achromobacter* (65.40%) and *Pedobacter* (8.36%). *Achromobacter* (55.07%) and *Pedobacter* (10.88%) dominated SC1_5. *Enterococcus* (51.43%) and *Carnobacterium* (27.32%) were the main genera in SC3, while *Enterococcus* (51.97%) and *Carnobacterium* (15.67%) dominated in SC6. Among the 463 identified genera across all skin groups, 87 genera significantly differed (KWH test, *p* < 0.05; Figure 5B and Figure S4).

The dominant phyla of the gut microbiota groups were Firmicutes, Proteobacteria, and Actinobacteria (Figure 5C). Among the 19 identified phyla, 12 phyla exhibited significant differences across the five gut groups (KWH test, *p* < 0.05; Figure 5C). *Eubacterium* (42.54%) and *Aurantimicrobium* (20.97%) were significantly enriched in Group SG0. *Eubacterium* (46.48%) and *Legionella* (17.90%) were characteristic of Group SG0_75. In Group SG1_5, *Eubacterium* (38.64%) and *Anaerocolumna* (9.62%) were dominant. Group SG3 was notably predominated by *Eubacterium* (25.46%) and *Alkaliphilus* (13.70%). Group SG6 dominated in *Eubacterium* (38.45%) and *Alkaliphilus* (14.51%) (Figure 5D). Among the 405 genera identified across the five gut groups, 177 genera significantly differed (KWH test, *p* < 0.05; Figure 5D).

### 3.6 Diversification of Skin and Gut Microbiota in Tadpoles

**FIGURE 6:**
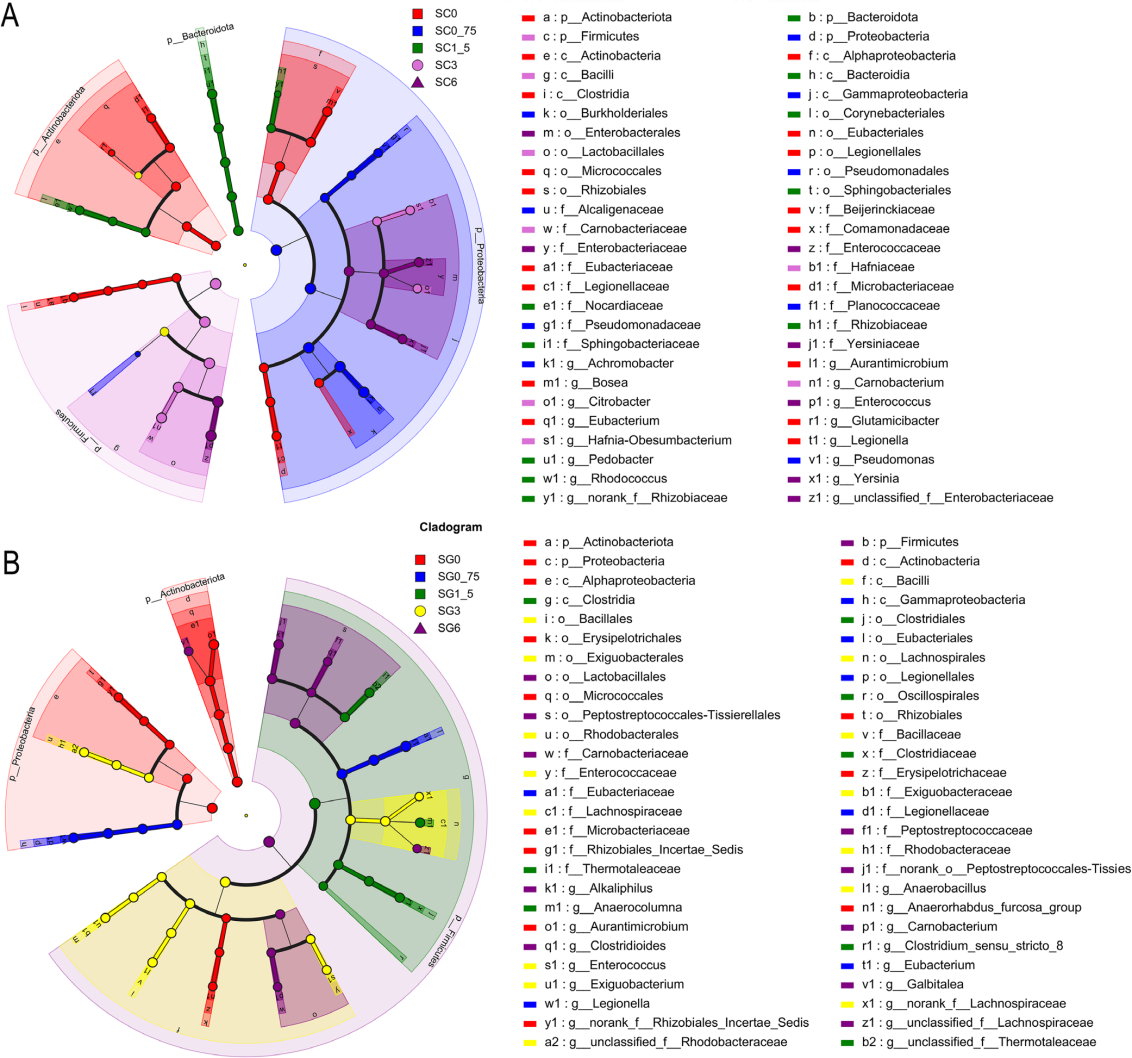
Investigating variations in tadpole skin and gut microbiota under five treatments of aqueous extracts of rice straw ash exposure using linear discriminant analysis effect size (LEfSe). The cladogram delineates the phylogenetic distinctions among microbiota taxa, highlighting the divergences in skin (A) and gut (B) microbiota among five concentrations (SC0, SG0, and SG3: *N* = 9; SC0_75, SC1_5, SC3, SC6, SG0_75, SG1_5, and SG6: *N* = 8). Color differentiation post‐treatment grouped samples into distinct categories. The relative abundance of each taxon is indicated by the size of the circles within the cladogram. Circle size denotes group abundances, while a detailed multiclass analysis reveals discrepancies across at least one class, delineating the taxonomic hierarchy from domain to genus via concentric circles. Labels for phylum through genus and taxa with LDA scores exceeding 4 are illustrated. The color‐coded legend on the right indicates the taxonomic classification of each node, ranging from phylum (p) to genus (g), and corresponds to the colors represented in the cladogram.

LEfSe analysis showed Actinobacteriota, Proteobacteria, Bacteroidota, and the Firmicutes were significantly enriched in groups SC0, SC0_75, SC1_5, and SC3, respectively (LDA > 4, *p* < 0.05; Figure 6A). *Aurantimicrobium, Bosea, Eubacterium, Glutamicibacter*, and *Legionella* were enriched in SC0; *Achromobacter* and *Pseudomonas* in SC0_75; *norank_f__Rhizobiaceae*, *Pedobacter*, and *Rhodococcus* in SC1_5; *Carnobacterium*, *Citrobacter*, and *Hafnia‐Obesumbacterium* in SC3; *Citrobacter*, *unclassified_f__Enterobacteriaceae*, and *Yersinia* in SC6 (LDA > 4, *p* < 0.05; Figure 6A).

LEfSe analysis revealed Actinobacteria and Proteobacteria were enriched in group SG0, and the abundance of Firmicutes significantly increased in group SG6 (LDA > 4, *p* < 0.05; Figure 6B). Other significant enrichments included *Anaerorhabdus_furcosa_group*, *Aurantimicrobium*, *norank_f__Rhizobiales_Incertae_Sedis* in SG0; *Eubacterium* and *Legionella* in SG0_75; *Anaerocolumna*, *Clostridium_sensu_stricto_8* in SG1_5; *Anaerobacillus*, *Enterococcus*, *Exiguobacterium, norank_f__Lachnospiraceae*, and *unclassified_f__Rhodobacteraceae* in SG3; *Alkaliphilus*, *Carnobacterium*, *Clostridioides*, *Galbitalea*, and *unclassified_f__Lachnospiraceae* in SG6 (LDA > 4, *p* < 0.05; Figure 6B).

### 3.7 BugBase Algorithm to Estimate Bacterial Phenotypes

**FIGURE 7:**
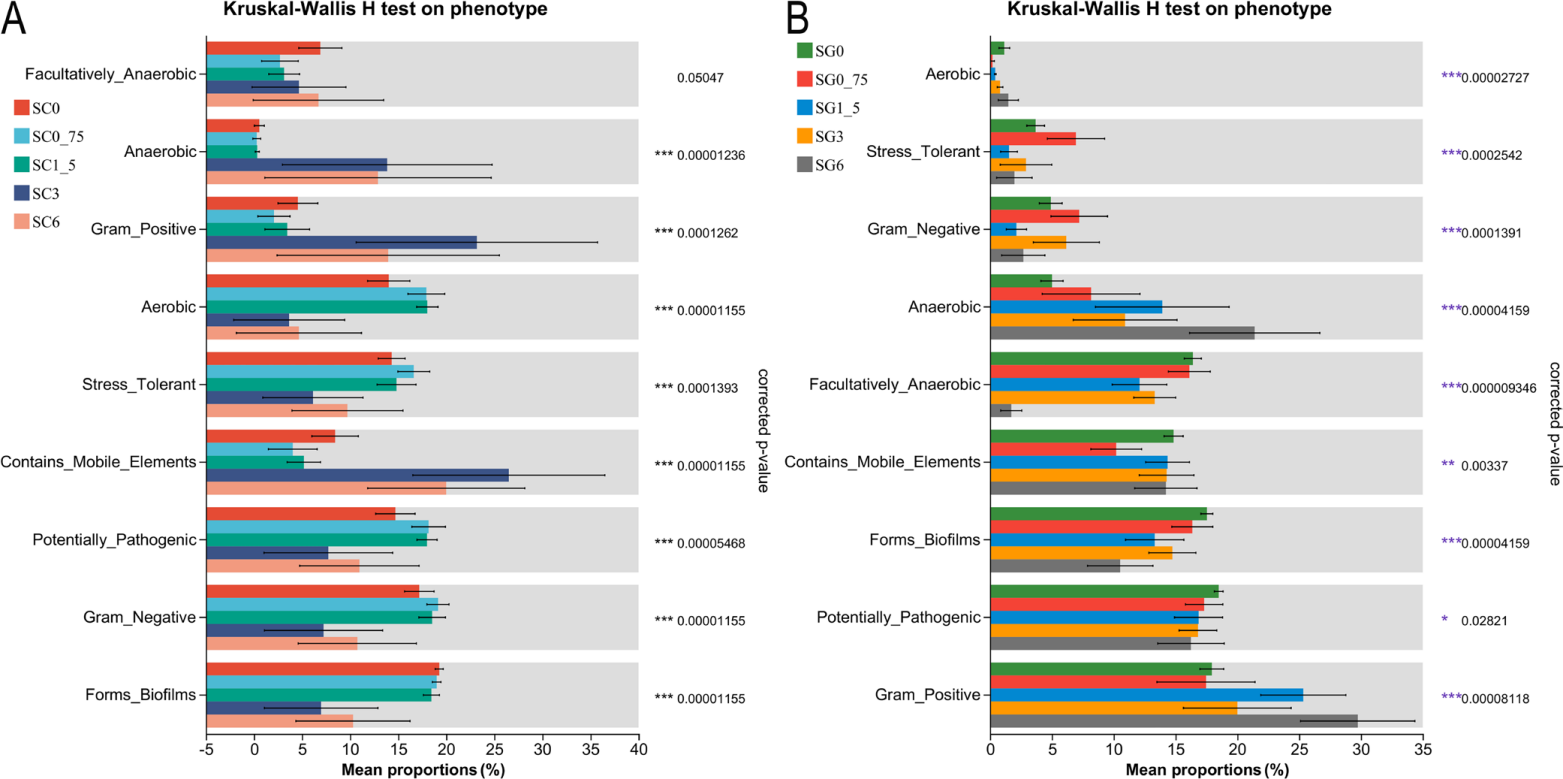
Using BugBase examined aqueous extracts of rice straw ash impact on *R. dybowskii* skin and gut microbiomes at various concentrations. Assessing the impact of aqueous extracts of rice straw ash at five concentrations (SC0, SG0, and SG3: *n* = 9; SC0_75, SC1_5, SC3, SC6, SG0_75, SG1_5, and SG6: *n* = 8) on the phenotype of skin (A) and gut (B) microbiota in tadpoles, using the BugBase approach to examine and predict bacterial characteristics. Significance levels are indicated: *, **, and *** for *p* values falling in 0.01 < *p* < 0.05, 0.001 < *p <* 0.01, and *p* < 0.001, respectively.

BugBase analysis revealed significant differences in eight phenotypes across the five skin groups, including Aerobic, Anaerobic, Contains_Mobile_Elements, Forms_Biofilms, Gram_Negative, Gram_Positive, Potentially_Pathogenic, and Stress_Tolerant (KWH test, *p* < 0.05, Figure 7A). The five skin groups did not significantly differ in Facultatively_Anaerobic phenotypes (KWH test, *p* > 0.05, Figure 7A). Analysis using BugBase on the five gut groups identified significant differences in nine phenotypes. These phenotypes included Aerobic, Anaerobic, Contains_Mobile_Elements, Facultatively_Anaerobic, Forms_Biofilms, Gram_Negative, Gram_Positive, Potentially_Pathogenic, and Stress_Tolerant (KWH test, *p* < 0.05, Figure 7B).

## 4 Discussion

### 4.1 PAHs From Ash: A Hidden Risk to Amphibians

This study is the first to identify that rice straw ash entering water bodies acts as an overlooked source of environmental PAHs pollution and warns that practices such as returning ash to fields may pose secondary pollution risks. This challenges the notion of biomass combustion as clean and underscores the need for improved agricultural pollution control and waste utilisation strategies. Notably, concentrations of ANT (1.52 × 10^−4^ μg mL^−1^) and FLT (1.29 × 10^−4^ μg mL^−1^) in AEA exceeded the European Union's environmental quality standards (1.00 × 10^−4^ and 1.20 × 10^−4^ μg mL^−1^, respectively) (European Union, 2013). In the present study, PAHs may reduce survival, delay development, and disrupt the gut and skin microbiota of *R. dybowskii* tadpoles through multiple pathways. Tadpoles' highly permeable skin allows lipophilic PAHs to infiltrate directly, and microbiota disruption may enhance this permeability (Llewelyn et al. 2019; Bilodeau et al. 2019). For example, early microbial disruption diminished adult frogs' capacity to resist parasites, indicating that microbiome integrity is essential for barrier function and immune maturation (Knutie et al. 2017). Tadpoles' gill‐based respiration, with thin structures and abundant blood flow, facilitates transdermal PAHs absorption (Chang et al. 2024). Moreover, ingesting contaminated water, particles, or algae introduces PAHs into the gut, where dysbiosis may enhance intestinal permeability and transform PAHs into more toxic derivatives (Zungum and Imam 2021; Leung et al. 2023; Wang et al. 2025). The accumulation of PAHs in sediments and low trophic level species contributes to indirect exposure and biomagnification through feeding and benthic activities (Bilodeau et al. 2019; Mai et al. 2024). For example, the PAHs in the bodies of *Lithobates sylvaticus* tadpoles exposed to sediment were markedly elevated compared to those in the water‐exposed group, signifying that sediment intake and contact were the main exposure routes (Bilodeau et al. 2019). In summary, PAHs in AEA harm tadpole development and micro‐ecological stability through multiple pathways, constituting an underestimated ecological risk.

### 4.2 Alpha Diversity and Beta Diversity

Our research identified substantial differences in alpha diversity indices across varying amounts of AEA in the environment of *R. dybowskii* . These fluctuations reflect a dramatic influence on microbial diversity throughout both skin and gut microbiota (Tong et al. 2023). Interestingly, group SG0 demonstrated lower microbial diversity compared to the ash‐exposed groups. Meanwhile, this discovery could point to the intricate relationships between microbial diversity and environmental stresses. One reason is the hormesis effect, which occurs when a little level of a stressor causes advantageous reactions in living things. In this situation, ash, even though it is a pollutant, may contribute to certain environmental stress that encourages a more varied microbial population (Hartmann and Six 2023). This result may be advantageous to the growth and well‐being of tadpoles up to a certain point. The chemical makeup of rice straw ash, which is rich in silica and perhaps trace metals, may change water chemistry, which can immediately impact microbial populations by promoting certain microbes while stifling others (Goodman 2020; Gay‐des‐Combes et al. 2017). These modifications to the microbiological environment may chronically affect the survival, growth, and well‐being of host organisms (Tong et al. 2023). Skin and gut microbiomes are important for the immune system function, pathogen defense, and nutrition intake of tadpoles (Miller et al. 2023). Changes in these microbiota may thus substantially impact the growing and surviving abilities of tadpoles, which may alter population dynamics and ecological functions (Jiménez and Sommer 2016). Future studies must address various limitations, even if our results provide insights into the ecosystem effects of agricultural wastes (Alan and Köker 2023). However, this research, limited to a controlled environment, may not properly manifest the complications of wild ecosystems, where results may be affected by interspecific interactions and varying factors (Naeem et al. 2012).

The Bray‐Curtis and weighted UniFrac distance, which revealed changes in gut microbiota structures across various levels of rice straw ash, clarify the remarkable effects of agricultural wastes on aquatic environments (Koul et al. 2022). We have studied how ash exposure impairs amphibian health and ecological balance; however, the types of ash (wildfire vs. rice straw), *R. dybowskii* developmental stages (adults vs. tadpoles), and microbiota, survival, and development consequences differ (Dong et al. 2024; Xu et al. 2024). The evident alterations in the microbiota structure imply even minor modifications to the surrounding conditions may considerably alter the microbial communities of water creatures, which might impact the health, growth, and survival of such organisms (Sehnal et al. 2021). Particularly, ash content is correlated to the composition of microbial populations (Gomez Isaza et al. 2022). Samples from the same group were gathered, and closer concentrations resulted in shorter distances, indicating rice straw ash dose‐dependently affects the gut microbiota of tadpoles. This dose dependence implies it is crucial to monitor and regulate the amount of agricultural waste entering water ecosystems, which will lessen their adverse environmental impacts (Vicente et al. 2022). Our research is focused on examining how ash affects the microbiota diversity, tadpole survival, and growth paths in water settings. In addition to complicating the study, the methodological use of an ash level gradient mimics real‐world situations where ash dispersed in aquatic systems naturally fluctuates (Gomez Isaza et al. 2022). This variation in ash content is directly related to alterations in the gut microbiota makeup of tadpoles, a part of ecological interactions that is still mostly unknown (Hughey et al. 2016).

### 4.3 Microbial Composition Altered by Straw Burning

This study revealed that the microbiota of *R. dybowskii* , especially in the skin, is highly sensitive to environmental changes, particularly changes due to rice straw ash exposure. Such susceptibility is highlighted by the significant microbial diversification and changes across various ash levels (Zhang et al. 2021). Actinobacteria, Proteobacteria, Bacteroidetes, and Firmicutes exist among all groups, indicating a complex microbial community that is sensitive to outside stimuli (Brito 2021). The enriched taxa in the control group (*Auerantimicrobium*, *Bosea*, *Eubacterium*, *Glutamicibacter*, and *Legionella*) point to a baseline diversity that is needed to preserve tadpole health (Jiménez et al. 2020). Moreover, the presence of *norank_f__Rhizobiaceae*, *Pedobacter*, *Rhodococcus*, and *Achromobacter* in group SC0_75 and *Pseudomonas* and *Achromobacter* in group SC1_5 suggests a shift toward bacteria that may play distinct roles in the physiological and immune responses of tadpoles. The presence of *Citrobacter* and *Yersinia* in the higher‐level groups (SC3 and SC6), known as opportunistic pathogens, raises concerns about the impact of ecological stresses on tadpole health and viability (McDaniel et al. 2007). For example, *Citrobacter* may infect animals and humans (Aguirre‐Sánchez et al. 2023), indicating that high levels of rice straw ash may pose risks to tadpole health and growth (Gao et al. 2019). The presence of *Yersinia*, particularly in the group with the highest ash level, includes some known pathogenic species and hints at potential bad consequences for tadpole health (Platt‐Samoraj 2022). These results uncover the vulnerability of amphibian microbiota to environmental variations, the ecological impact of agricultural practices (e.g., stubble burning) on water environments and their inhabitants, and the functions of microbiota in host health, including growth and disease defense (Bernardo‐Cravo et al. 2020).

### 4.4 Functional Prediction Analysis of Skin and Gut Microbiota

The observed phenotypic changes suggest a dynamic microbial adaptability to environmental stressors, which can significantly affect host‐microbe symbiosis (Voolstra and Ziegler 2020). These phenotypic discrepancies emphasize the complicated connections that constitute microbial community structure and functions, reflecting how they adapt to environmental stresses (Scheuerl et al. 2020). This study uncovers considerable discrepancies in skin and gut defense phenotypes across testing groups. These findings indicate that microbial communities are vulnerable to diverse environmental stressors and that the changes will bring potential health effects on microbe composition and function (Hernandez et al. 2021). The identified phenotypic discrepancies indicate that microbes dynamically adapt to environmental stressors, which potentially affect host‐microbe symbiosis (Voolstra and Ziegler 2020). Phenotypic changes show how intricate connections impact microbe community structure and functions, highlighting their adaptation to surrounding constraints (Pacheco et al. 2021). The ability for biofilm formation, implied by the Forms_Biofilms phenotype, indicates an extra layer of microbiome protection and connection with the host, improving microbial pathogenicity and resilience (Rather et al. 2021). The considerable change in these phenotypes among treatment groups uncovers the complication of microbial reactions to external stimuli and brings key questions about the influence of this diversity on host health and diseases. For example, the balance between Gram_Positive and Gram_Negative bacteria as well as the ratio of Potentially_Pathogenic species could directly impact the host's immune reactions, infection susceptibility, and overall health conditions (Pickard et al. 2017). This study calls for more exploration into the procedures and host‐microbiota impacts of these phenotypic variations. How pollutants and other stressors affect the microbiota phenotypes shall be further studied (Jin et al. 2017). Clarifying these processes is crucial for managing microbe populations to enhance host health and mitigate disease risk (Trevelline et al. 2019).

### 4.5 Body Mass, Survival Rate and Development Stage

In this study, tadpoles in low ash groups (S0 and S0_75) were larger than those in higher ash groups (S1_5, S3 and S6), indicating that low levels of ash can boost growth up to a certain limit. However, high ash concentrations significantly reduced survival rates, particularly in S6, and delayed development, showing a clear dose‐dependent effect on tadpole physiology. This underscores the importance of understanding the dose‐dependent relationship between ash content and tadpole health to assess the ecological impacts of agricultural waste disposal (Hughey et al. 2016). High ash levels can harm tadpole growth and survival due to the presence of toxic chemicals and the physical characteristics of ash particles. Our previous study found that rice straw ash and its AEA contain significant amounts of potassium, calcium, and magnesium, as well as detectable levels of heavy metals such as arsenic and copper, all of which pose potential threats to amphibian survival (Dong et al. 2024). These elements can induce electrolyte imbalance, oxidative stress, and enzymatic disruption, thereby impairing physiological functions and increasing mortality in amphibians (Voyles et al. 2009; Brix et al. 2022). Multiple PAHs were found in AEA, which, along with heavy metals, are known to negatively affect the health and growth of aquatic species due to their chemical properties (Barathi et al. 2023; Maletić et al. 2019). Rice straw ash particles, with their porous structure, can adsorb these harmful substances and release them into the aquatic environment, further affecting tadpole health. The irregular shapes and sizes of ash particles may also physically damage the skin and gills of tadpoles, interfering with their respiration and nutrient uptake (Cao et al. 2022). Furthermore, even small environmental changes can have a significant impact on the tadpole microbiota, as indicated by the Bray‐Curtis distance and weighted UniFrac distance metrics. Changes in microbiota composition can impair immune function, nutrition intake, and overall fitness of tadpoles (Zhu et al. 2023). Our study observed considerable changes in microbiota composition, which negatively affected tadpole growth, development, and survival, emphasizing the need for careful monitoring and control of agricultural waste products to protect aquatic health (Fontaine et al. 2022).

### 4.6 Straw Burning Impacts and Management Strategies

Straw burning is a widespread agricultural practice, but the annual burning of over 100 million tons of straw globally poses a significant threat to biodiversity (Singh, Sharma, et al. 2024). Our study indicated that rice straw ash (containing PAHs) enters wetland ecosystems and disrupts the balance of skin and gut microbiota in tadpoles, suppresses beneficial microbes, promotes pathogenic bacteria, impairs development, and reduces survival. Therefore, the conventional burning of straw constitutes a potential global ecological threat. To protect wetland ecosystems and global biodiversity, it is necessary to implement synergistic measures across the following aspects. Advocate for agronomic alternatives such as returning appropriately crushed straw to the field to achieve a secure nutrient cycle (Wang et al. 2024). Enhance environmental consciousness, strengthen agricultural education, and guide farmers in thoroughly understanding the detrimental effects of burning on the ecological environment, particularly concerning amphibians (Despotović et al. 2021). Formulate scientific policies informed by the characteristics of farmland and the empirical findings presented in this study, and implement tailored straw management policies (such as return‐to‐field criteria) to minimize ecological risks within the agricultural management system (Li et al. 2023). Furthermore, an ecological monitoring and early warning system should be established to continually track the pollution status of PAHs in wetlands and farmland, monitor alterations in microbial communities, and biological responses, so enabling quick detection and control of ecological risks (Klimkowicz‐Pawlas et al. 2019). Encourage deep collaboration among diverse disciplines, including environmental science, microbiology, and agronomy, to thoroughly investigate ecotoxicological mechanisms, optimize return‐to‐field technologies, and accelerate the translation of scientific advancements into practical and effective protective measures (Vaverková et al. 2024). These comprehensive measures can more extensively and scientifically address the ecological challenges presented by straw burning.

## 5 Conclusions

The study demonstrates that aqueous rice straw AEA includes PAHs, the ash particles possess an irregular porous structure, and different concentrations of AEA treatment have a dose‐dependent inhibitory effect on tadpole survival rate, body mass, and developmental progression. Ash exposure significantly altered the diversity and composition of skin and gut microbiomes, with a significant enrichment of potential pathogenic bacteria in the high‐concentration treatment group. BugBase predictions indicate that microbial communities are experiencing alterations in functional phenotypes. While conducted under controlled conditions, the findings offer valuable insights into the ecotoxicological risks posed by straw burning residues. Future research should occur in semi‐natural ecosystems over extended periods, and a priority on developing coordinated solutions for pollution reduction and ecological restoration to address biodiversity loss. This study provides a scientific basis for assessing the risks to freshwater amphibians impacted by pollution from farmland straw burning ash, creates new perspectives for comprehending environmental disturbances from macrohabitat to microsymbiotic levels, and establishes a paradigm for multi‐species, multi‐scale ecotoxicological evaluation and the design of ecological restoration strategies.

## Author Contributions

**Qing Tong:** conceptualization (lead), data curation (equal), methodology (equal), software (equal), writing – original draft (equal). **Yue‐liang Pan:** data curation (equal), investigation (equal), methodology (equal), software (equal), supervision (equal), writing – original draft (equal). **Qiu‐ru Fan:** data curation (equal), investigation (equal), methodology (equal). **Wen‐jing Dong:** data curation (equal), investigation (equal), methodology (equal), software (equal), writing – original draft (equal). **Xin‐zhou Long:** data curation (equal), investigation (equal), methodology (equal), software (equal), writing – original draft (equal). **Ming‐da Xu:** formal analysis (equal), investigation (equal), methodology (equal), software (equal), supervision (equal), writing – original draft (equal). **Li‐yong Cui:** data curation (equal), investigation (equal), methodology (equal), software (equal), writing – original draft (equal). **Zhi‐wen Luo:** conceptualization (equal), data curation (equal), formal analysis (equal), investigation (equal), software (equal), writing – original draft (equal).

## Ethics Statement

All animal protocols received approval from the Institutional Animal Care and Use Committee of Jiamusi University (IACUC#2020–035). All experiments were conducted in compliance with the established rules and regulations. All animal trials adhered to the principles of the 3 Rs (Replacement, Reduction, and Refinement) to avoid excessive and unnecessary sacrifice.

## Consent

The authors have nothing to report.

## Conflicts of Interest

The authors declare no conflicts of interest.

## Supporting information


**Appendix S1.** Supporting Information.

## Data Availability

(A) PRJNA1050065: tadpole skin microbiota of control group (TCC group) (0) https://www.ncbi.nlm.nih.gov/bioproject/PRJNA1050065. PRJNA1058650: tadpole skin microbiota of straw ash (SC0_75group) (0.75) https://www.ncbi.nlm.nih.gov/bioproject/PRJNA1058650. PRJNA1058652: tadpole skin microbiota of straw ash (SC1_5 group) (1.5) https://www.ncbi.nlm.nih.gov/bioproject/PRJNA1058652. PRJNA1058654: tadpole skin microbiota of straw ash (SC3 group) (3) https://www.ncbi.nlm.nih.gov/bioproject/PRJNA1058654. PRJNA1050068: tadpole skin microbiota of straw ash (TSC group) (6) https://www.ncbi.nlm.nih.gov/bioproject/PRJNA1050068. (B) PRJNA1050064: tadpole gut microbiota of control group (TCG group) (0) https://www.ncbi.nlm.nih.gov/bioproject/PRJNA1050064. PRJNA1058657: tadpole gut microbiota of straw ash (SG0_75 group) (0.75) https://www.ncbi.nlm.nih.gov/bioproject/PRJNA1058657. PRJNA1058658: tadpole gut microbiota of straw ash (SG1_5 group) (1.5) https://www.ncbi.nlm.nih.gov/bioproject/PRJNA1058658. PRJNA1058659: tadpole gut microbiota of straw ash (SG3 group) (3) https://www.ncbi.nlm.nih.gov/bioproject/PRJNA1058659. PRJNA1050067: tadpole gut microbiota of straw ash (TSG group) (6) https://www.ncbi.nlm.nih.gov/bioproject/PRJNA1050067.
