# Supplementary material for: Exposure to Rice Straw Ash Alters Survival, Development and Microbial Diversity in Amphibian Tadpoles
Source: Ecol Evol. 2025 Jul 25;15(7):e71801. doi: 10.1002/ece3.71801 (PMC12290308; doi:10.1002/ece3.71801)
Supplement: Supplementary file 1 — Appendix S1. Supporting Information. [file ECE3-15-e71801-s001.docx]

**Supplementary Material**

**Exposure to Rice Straw Ash Alters Survival, Development and Microbial Diversity in Amphibian Tadpoles**

Qing Tong^a, b^, Yue-liang Pan^a^, Qiu-ru Fan^a^, Wen-jing Dong^a^, Xin-zhou Long^a^, Ming-da Xu^a^, Li-yong Cui^b^, Zhi-wen Luo^a^**^*^**

^a^School of Biology and Agriculture, Jiamusi University, Jiamusi, 154007, China

^b^Jiamusi Branch of Heilongjiang Academy of Forestry Sciences, Jiamusi, 154002, China

**^*^Corresponding author**

Zhi-wen Luo (jmslzw@126.com)

Tel.: +86-454-5602255

Fax: +86-454-5602255

Word count: 6500

Number of figures: 7


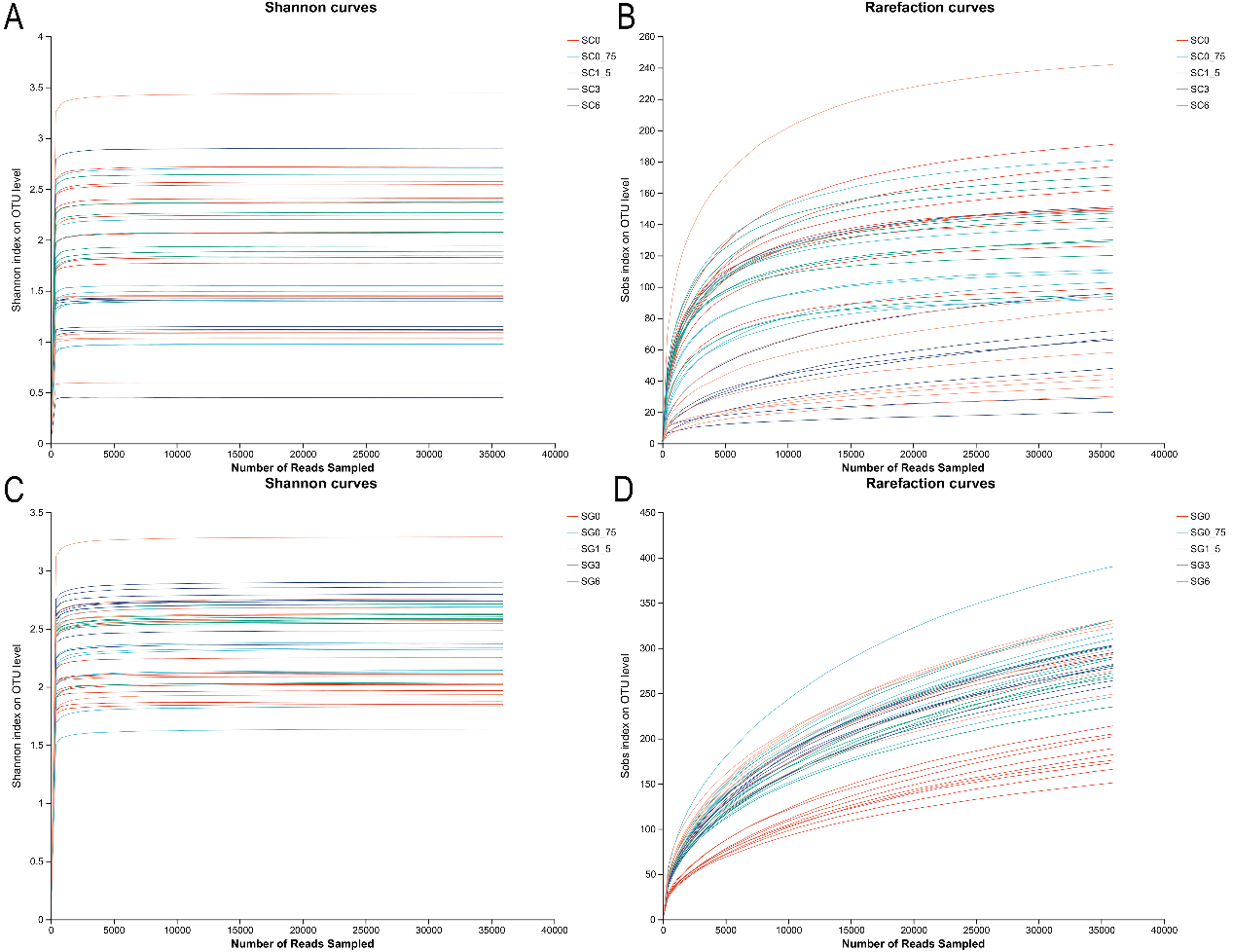


**Figure S1** Analyzing Shannon indices and rarefaction.

The Shannon and rarefaction curves graphically illustrate the relationship among the number of sequences and the corresponding variation in OTU counts, demonstrating how OTU diversity changes with sequencing depth. The Shannon curve reflects the skin and gut microbiota diversity of the samples.


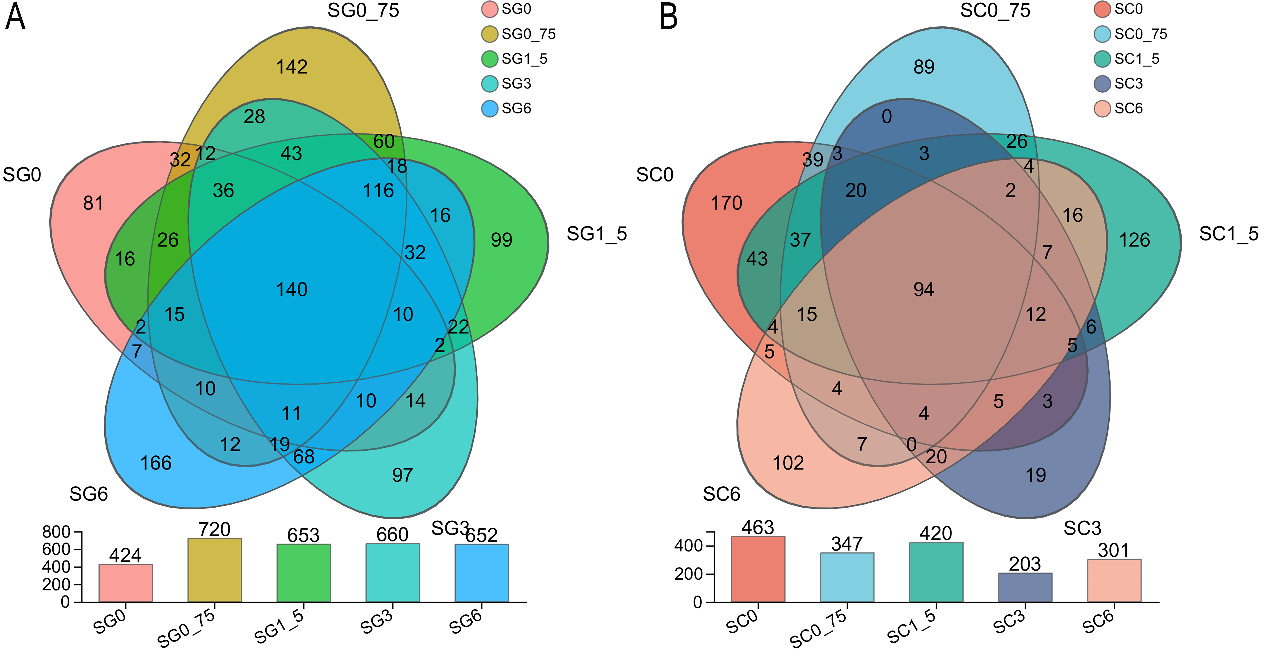


**Figure S2** Venn diagram displays shared OTUs within skin and gut microbiomes across varying ash concentrations.

In the Venn diagram, the values within the intersecting areas of the three circles represent the number of OTUs shared among the three groups. The values adjacent to these intersections indicate the number of unique OTUs. Each group is represented by circles of different colors.


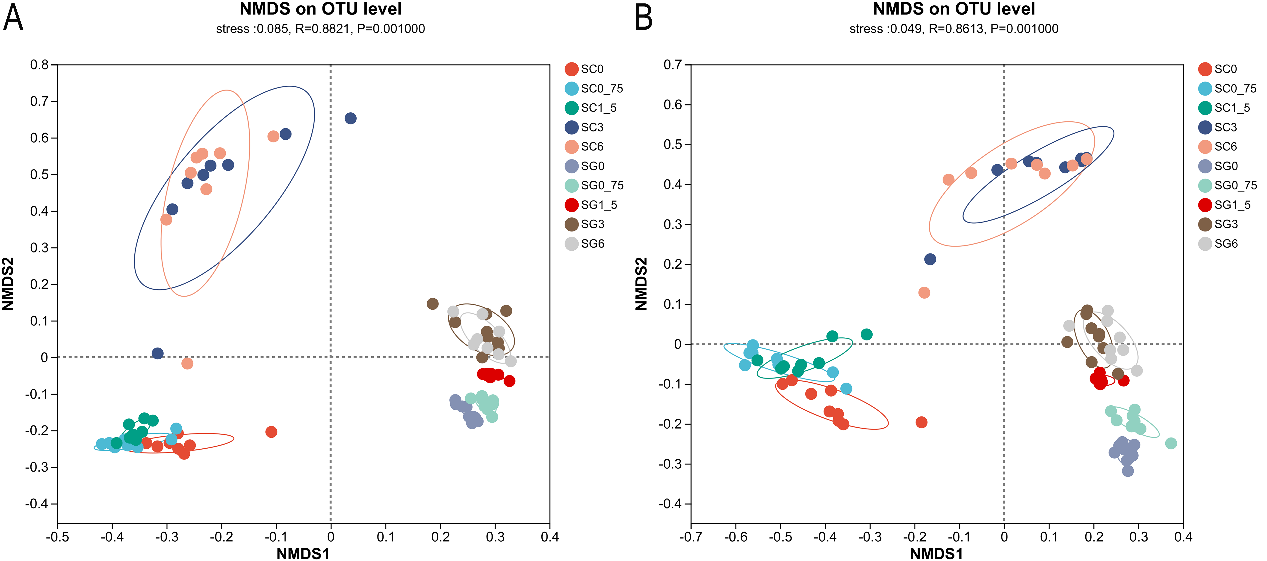


**Figure S3** Non-metric multidimensional scaling (NMDS) analysis into the impact of straw ash on the microbiota diversity of tadpoles.

Through NMDS plots employing Bray-Curtis (A) and weighted UniFrac (B) distances, the divergence among communities is depicted. The plots position each sample based on the microbial composition of either skin or gut, where the spatial closeness denotes group similarities. The significance is determined by *P*-values from Adonis and ANOSIM tests.


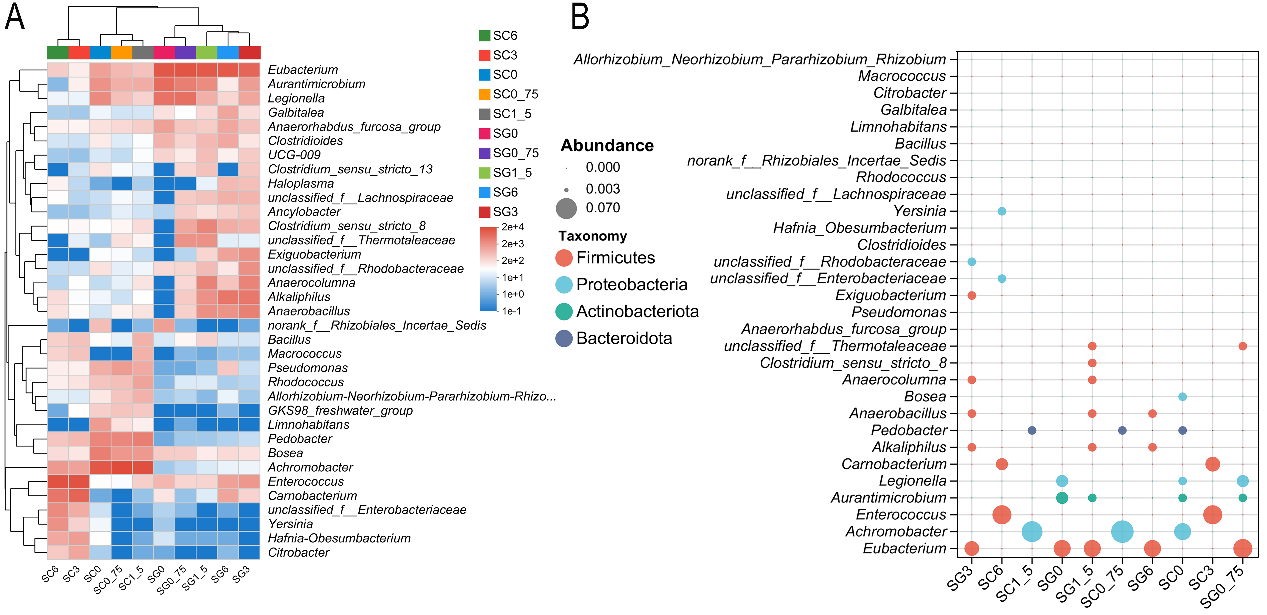


**Figure S4** Heatmap depicting the genus-level differences across groups treated with varying concentrations of straw ash.

Using Bray-Curtis distances and the average linkage method, the cluster analysis assigns each sample to either a bar or column. Log-transformed relative abundance data (A) are visualized on a gradient from blue to red, where blue signifies lower and red signifies higher values (B).
